# Ecological momentary assessment of targeted and non‐targeted symptom changes in clinical anxiety and irritability among children and adolescents receiving cognitive behavioral therapy

**DOI:** 10.1002/jcv2.70160

**Published:** 2026-08-30

**Authors:** Lauren M. Henry, Meghan E. Byrne, Julia Modell, Lili Massac, Krystal Lewis, Simone Haller, Reut Naim, Katharina Kircanski, Daniel S. Pine, Melissa A. Brotman

**Affiliations:** ^1^ Emotion and Development Branch National Institute of Mental Health (NIMH) Bethesda Maryland USA; ^2^ School of Psychological Sciences Tel‐Aviv University Tel‐Aviv Israel; ^3^ Sagol School of Neuroscience Tel‐Aviv University Tel‐Aviv Israel; ^4^ Department of Psychology University of Southern California Los Angeles California USA

**Keywords:** anxiety, cognitive behavioral therapy, ecological momentary assessment, irritability, transdiagnostic, youth

## Abstract

**Background:**

Decades of youth psychotherapy research have demonstrated the efficacy of cognitive behavioral therapy (CBT) on targeted symptoms using standardized, retrospective clinician‐, child‐, and parent‐report. Advancements in digital technologies can be leveraged to capture granular symptom change in patients' natural environments. While CBT is tailored to treat specific diagnosis‐perpetuating mechanisms, it remains unknown whether such effects may carry over to non‐targeted symptoms, which could have important treatment implications.

**Methods:**

We examined targeted and non‐targeted symptom change in two separate exposure‐based CBT trials: one trial for anxiety (*n* = 92) and one for irritability (*n* = 39). Youth in both trials completed ecological momentary assessment (EMA) pre‐, mid‐, and post‐treatment; *n* = 57 healthy volunteer youth completed EMA at matching time intervals. Each EMA interval lasted for 7 days (3 prompts/day). We used multilevel modeling to examine changes in symptoms across time (e.g., pre, mid, post).

**Results:**

We observed frustration to decrease over time in both youth with anxiety (non‐targeted symptom) and irritability (targeted symptom). We observed anger to decrease in youth with irritability only (targeted symptom), and worry did not change significantly over time in either group. Healthy volunteer youth showed low, stable levels of symptoms across time.

**Conclusion:**

Findings highlight frustration as a potential key transdiagnostic symptom; frustration improves in both youth with clinical anxiety and with irritability over the course of exposure‐based CBT. Demonstrating specificity, anger in youth seeking treatment for irritability, but not anxiety, improved over time. While additional research is needed to better understand measurement of in vivo worry, results support using EMA to monitor symptom change during CBT.

## Key Points

**What's known?**

- While cognitive behavioral therapy (CBT) is efficacious for targeted symptoms in youth with anxiety and irritability, it is unclear whether effects generalize to non‐targeted symptoms in daily life.

**What's new?**

- Using ecological momentary assessment (EMA), we observed non‐targeted frustration to decrease for youth receiving CBT for anxiety. Targeted worry did not change for youth with anxiety, and this finding may relate to measurement limitations.

**What's relevant?**

- Frustration may be further examined as a transdiagnostic treatment target for both pediatric anxiety and irritability. Additional research is needed to understand and improve the in vivo measurement of anxiety.

## INTRODUCTION

Cognitive behavioral therapy (CBT) has been adapted to treat various psychiatric disorders in youth (Crowe & McKay, 2017; Hofmann et al., 2012). Multiple randomized controlled trials have demonstrated that CBT is efficacious and effective in treating targeted symptoms among youth receiving treatment for anxiety (James et al., 2020; Peterson et al., 2026; Warwick et al., 2017), and early studies suggest efficacy for pediatric irritability (Naim et al., 2025). When treating a particular disorder, CBT effects may carry over to non‐targeted symptoms; for example, reductions in depressive and externalizing symptoms have been observed in children receiving CBT for anxiety disorders (Kreuze et al., 2018). This phenomenon is particularly relevant for pediatric anxiety and irritability, which are highly comorbid (Leibenluft, 2011; Stoddard et al., 2014). Given the persistent mismatch between the need for and availability of treatment, determining whether aspects of symptom change during CBT for youth anxiety and/or irritability may be transdiagnostic has critical real‐world implications (Marchette & Weisz, 2017). Still, generalizability versus specificity in CBT symptom targets, especially with regard to anxiety and irritability, has not been well explored (Schaeuffele et al., 2021). Here, we investigate and compare changes in child‐rated in vivo symptoms over the course of two CBT treatment protocols—one targeting anxiety and the other targeting irritability—to determine whether these treatments translate to non‐targeted symptoms.

Anxiety and irritability are related yet distinct. Both are characterized by heightened negative affectivity and biased attention toward cues perceived as threatening or aversive (Kircanski et al., 2018). They differ in their central emotional states and typical behavioral responses. Fear (central to anxiety) and anger (central to irritability) are both acute, high‐arousal, and typically stimulus‐driven emotional states. However, fear is associated with a maladaptive avoid response in anxious youth, and anger with a maladaptive approach response (e.g., temper outbursts) in irritable youth (Carver & Harmon‐Jones, 2009; Tarpley et al., 2010). Exposure is a therapeutic technique to confront stimuli or situations that evoke feelings of fear or anger and decrease the associated maladaptive responses (Knowles & Tolin, 2022). In the current research, we use two theoretically overlapping exposure‐based CBT manuals. Our CBT for anxiety manual (Silverman, 2017) uses exposures to target aberrant responses (e.g., avoidance) to fear‐inducing stimuli, and our CBT for irritability manual (German et al., 2026) uses exposures to target aberrant responses (e.g., approach via temper outbursts) to anger‐inducing stimuli. In both manuals, therapist and patient construct and progress through a graded hierarchy of in vivo exposures to fear or anger triggers, respectively. These treatments may affect symptom change through similar pathways (e.g., emotion regulation, inhibitory learning; Craske et al., 2014; Knowles & Tolin, 2022). Accordingly, both targeted and non‐targeted symptoms may change in response to treatment. That is, CBT for anxiety may also be associated with reduced anger/annoyance and frustration, and CBT for irritability with reduced fear/worry.

Prior CBT research has predominantly used standardized, retrospective child‐ and parent‐report (Sigurvinsdóttir et al., 2020) or clinician ratings (Miguel et al., 2025; Moskowitz & Young, 2006) to examine symptom change across treatment. However, retrospective reports may be influenced by memory recall biases, and neither retrospective reports nor clinician ratings can capture granular changes in psychopathology symptoms across time and context (Walz et al., 2014). Advances in digital technologies can be leveraged to address the limitations of these other rating methods. Ecological momentary assessment uses repeated sampling to capture real‐time changes in symptoms in patients' natural environments. By repeatedly sampling patients' in vivo psychological states, EMA reduces recall bias, promotes ecological validity, and allows researchers to capture within‐person symptom variability and their lagged relations (Ebner‐Priemer & Trull, 2009; Shiffman et al., 2008). Relatedly, in treatment research, EMA variables have captured unique variance beyond more traditional reports (e.g., self‐report questionnaires, clinical interviews) in models predicting response and dropout, symptom recurrence, and diagnostic status (Mink et al., 2025). Compared with traditional questionnaire‐based assessments, EMAs may also facilitate a more straightforward transition to digital interventions. That is, ecological momentary interventions delivered in response to symptom occurrence in daily life may support progress toward precision medicine.

In the current study, we assess EMA‐reported changes in targeted and non‐targeted symptoms before, during, and after CBT for children and adolescents seeking treatment for an anxiety disorder diagnosis. We compare our results to previously published findings from a parallel EMA protocol of symptom changes throughout CBT targeting pediatric irritability (Naim et al., 2026). By leveraging EMA's ecological precision, we aim to clarify how targeted and non‐targeted symptoms change over the course of CBT in the everyday lives of youth. Given the high comorbidity of anxiety and irritability in pediatric patients, our use of parallel, symptom‐focused EMA protocols across these distinct but interrelated clinical samples allows for investigation of both treatment‐targeted symptom change, as well as change in corresponding, non‐targeted, yet highly co‐occurring symptoms (Crum et al., 2020). We hypothesize that anxiety patients will exhibit reductions in targeted symptoms corresponding to their primary diagnoses (EMA‐reported fear/worry). We hypothesize that non‐targeted symptoms will also decrease, such that youth treated for anxiety will also report reductions in irritability symptoms (EMA‐reported anger/annoyance and frustration). Finally, we include a group of healthy volunteers as a descriptive comparator to support interpretation of EMA‐reported symptoms in the clinical samples. We hypothesize that healthy volunteers will report lower levels of symptoms at baseline than youth with anxiety or irritability and that these reports will remain low and stable across time.

## METHODS

All procedures were approved by the National Institute of Mental Health Institutional Review Board. All parents provided written informed consent, and all children provided written assent, for participation. For further methodological details (e.g., eligibility, samples, treatment), see Byrne et al. (2023) for the anxiety and healthy volunteer samples, and see Naim et al. (2025, 2026) and Naim, Kircanski, et al. (2021) for the irritability sample.

### Participants

#### Recruitment and overall sample

Youth ages 8–18 years were recruited from the greater Washington, DC metropolitan area to participate in the larger study protocols. Three groups of participants (total *N* = 188) were included in the current study: (1) medication‐free youth seeking treatment for an anxiety disorder (e.g., generalized, social, or separation anxiety disorder) who did not meet criteria for an ongoing mood disorder, (2) youth diagnosed with clinically impairing irritability (e.g., disruptive mood dysregulation disorder; DMDD), and (3) healthy volunteer youth with no psychiatric diagnosis. See Table 1 for demographic and diagnostic descriptives and Supporting Information S1: Appendix S1 for comorbidities.

**TABLE 1:** Demographic characteristics of study participants.

	Irritability (*n* = 39)	Anxiety (*n* = 92)	Healthy volunteers (*n* = 57)
Age at baseline assessment, *M* (SD)	11.26 (1.89)	12.78 (2.70)	12.97 (2.74)
Sex (%)			
Female	35.90	61.96	54.39
Male	64.10	38.04	45.61
Ethnicity (%)			
Hispanic or Latino/a	5.13	17.39	3.51
Not Hispanic or Latino/a	89.74	78.26	96.49
Unknown	5.13	4.35	0.00
Race (%)			
American Indian/Alaska Native	0.00	1.09	0.00
Asian	2.56	4.35	3.51
Black or African American	10.26	2.17	17.54
White	79.49	68.48	57.89
More than one race	5.13	18.48	19.30
Other/Unknown	2.56	5.43	1.75
Primary diagnosis (%)^a^	61.54 DMDD, 25.64 ODD, 12.82 ADHD	100.00 anxiety^b^	None

*Note*: Given age and sex differences between samples, supplementary multilevel model results adjusting for age and sex covariates are included in Supporting Information S1: Table S2.

Abbreviations: ADHD, attention‐deficit/hyperactivity disorder; DMDD, disruptive mood dysregulation disorder; ODD, oppositional defiant disorder.

^a^ See Supporting Information S1: Appendix S1 for details on comorbidity.

^b^ All youth met criteria for at least one primary anxiety disorder: sixty‐seven (72.83%) with generalized anxiety disorder, 58 (63.04%) with social phobia, 24 (26.09%) with separation anxiety disorder, 14 (15.22%) with agoraphobia, 3 (3.26%) with panic disorder, and 2 (2.17%) with selective mutism.

#### Anxiety sample

On average, youth with anxiety (*n* = 92) were 12.78 years old. The sample consisted of 61.96% female participants, 68.48% youth who identified as White, and 78.26% who identified as Non‐Hispanic or Latino. All youth met criteria for at least one primary anxiety disorder.

#### Irritability sample

On average, youth with irritability (*n* = 39) were 11.26 years old. The sample consisted of 64.10% male participants, 79.49% youth who identified as White, and 89.74% who identified as Non‐Hispanic or Latino. All youth met criteria for clinically impairing irritability: 24 (61.54%) for primary DMDD, 10 (25.64%) for primary oppositional defiant disorder (ODD), and 5 (12.82%) for primary attention‐deficit/hyperactivity disorder (ADHD).

#### Healthy volunteer sample

On average, healthy volunteers (*n* = 57) were 12.97 years old. The sample consisted of 54.39% female participants, 57.89% youth who identified as White, and 96.49% who identified as Non‐Hispanic or Latino. All youth were determined to have no current or lifetime psychiatric diagnosis.

### Procedure

#### Eligibility

Eligibility was determined in part by psychiatric diagnostic status based on the primary presenting problem. All youth in the anxiety sample were determined to have a current anxiety disorder as their primary presenting diagnosis, and all youth in the irritability sample were determined to have current DMDD as their primary presenting diagnosis or a presenting problem characterized by severe and impairing irritability (i.e., via temper outbursts or irritable mood) in more than one domain (home, school, peers). No healthy volunteer youth met criteria for a current or lifetime psychiatric disorder.

#### Treatment

Both the anxiety and irritability samples were administered 12 sessions of exposure‐based CBT. For the anxiety sample, exposures targeted anxiety (Silverman, 2017), and for the irritability sample, exposures targeted irritability (German et al., 2026). A subset of the anxiety sample also participated in a randomized controlled trial in which they were randomly assigned to either an active or sham condition of an augmentative computer‐based attention bias modification training (ABMT). All parents in the irritability sample also received therapist‐delivered parent management training (PMT). Healthy volunteers received no treatment.

For anxiety, CBT sessions lasted between 40 and 60 minutes, followed by additional home practice assignments to build on exposures completed during therapy. The first three sessions consisted of an introduction to CBT, self‐monitoring, and psychoeducation. Patients were instructed to complete their first out‐of‐session exposure after the third week, followed by in‐session exposures and cognitive restructuring beginning in week four. Emphasis was placed on increasing patient awareness of their anxious thinking patterns and learning more adaptive cognitive strategies.

For the irritability sample, sessions lasted between 60 and 120 minutes (30–60 minutes devoted to the child and to the parent). Child sessions included motivational interviewing, treatment goal setting, psychoeducation on irritability, development of an anger hierarchy, and progression from role plays and imaginal exposures to in vivo anger exposures with anger (“temperature”) ratings. During in vivo exposures, patients were taught to tolerate negative affect while withholding their prepotent approach behavioral response (i.e., temper outburst). Parent sessions included motivational interviewing, psychoeducation on irritability and instrumental learning, parent distress tolerance, and skill building around praise and rewards, active ignoring, effective commands, limit setting, and pleasant parent‐child interactions.

#### EMA

Symptoms related to anxiety and irritability were measured via smartphone in three 1‐week intervals at pre‐treatment, mid‐treatment, and post‐treatment. In CBT for anxiety, EMA was administered prior to the beginning of exposures at session 2 (pre‐treatment), between sessions 7 and 8 (mid‐treatment), and between sessions 11 and 12 (post‐treatment). In CBT for irritability, EMA was administered prior to CBT session 1 (pre‐treatment), between sessions 6 and 7 (mid‐treatment), and after session 12 (post‐treatment). For the healthy volunteer group, the three intervals occurred approximately 5 weeks apart after consenting to parallel the time intervals in which the patient groups completed EMA. All youth were prompted three times per day on a semirandom schedule for 7 consecutive days. Thus, participants were administered 21 prompts per interval, or 63 prompts total. The EMA protocol was identical at each treatment phase.

Research assistants trained participants to complete the EMA protocol (e.g., phone use, items) before the pre‐treatment EMA interval. Participants who did not have their own smartphone were provided with a loaner smartphone. During the training, participants selected three blocks of time for each of the 7 days during which to receive prompts. Specifically, participants selected 1‐h time blocks in the morning/before school (between 6:00 and 9:00 am), afternoon/after school (between 3:00 and 6:00 pm) and evening/before bedtime (between 7:00 and 10:00 pm). The exact time of prompt delivery was randomized within each selected 1‐h time block. Participants were given 1 hour to complete each prompt before the survey expired.

We used the Real Time Assessment in the Natural Environment (ReTAINE) to prompt participants. ReTAINE is a web‐based system developed by researchers at the Neuropsychiatric Research Institute in Fargo, North Dakota. At each prompt, participants received a text message with a link to the website where the EMA items were delivered (http://retaine.org/). In addition to receiving $90/interval to participate in the EMA protocol, participants were incentivized to complete as many prompts as possible through a $10 bonus for completing over 75% of prompts at each interval.

In line with best practices in EMA research, see Supporting Information S1: Table S1 for our comprehensive reporting of EMA features using the Adapted STROBE Checklist for Reporting EMA Studies (CREMAS; Liao et al., 2016).

### Measures

#### Psychiatric diagnoses

Licensed clinicians administered the Schedule for Affective Disorders and Schizophrenia for School‐Age Children—Present and Lifetime version (K‐SADS‐PL; Kaufman et al., 1997), including the DMDD module (Wiggins et al., 2016), to parents and children. The K‐SADS‐PL is a semistructured diagnostic interview that assesses present and lifetime disorders based on the Diagnostic and Statistical Manual of Mental Disorders, 5th Edition (American Psychiatric Association, 2013). Diagnoses were confirmed by a senior psychiatrist (Daniel S. Pine, MD for the anxiety sample and Lisa M. Cullins, MD for the irritability sample). Clinicians were trained to achieve acceptable reliability (κ > .60) with expert diagnosticians.

#### Psychological symptoms

To examine psychological symptoms of anxiety and irritability at baseline, two questionnaire measures (child/self and parent forms) were completed.

The Screen for Anxiety and Related Disorders (SCARED; Birmaher et al., 1997, 1999) is a 41‐item questionnaire used to assess anxiety symptoms in children and adolescents over the past three months. The reliability and validity of the SCARED have been demonstrated in previous research (e.g., Birmaher et al., 1999; Monga et al., 2000). In the current research, we administered both the Child Report (SCARED‐C) and Parent Report (SCARED‐P) forms. Items are rated on a three‐point scale from 0 (*Not True* or *Hardly Ever True*) to 2 (*Very True* or *Often True*). Responses to each item are summed to generate a total score ranging from 0 to 82. While not a standalone diagnostic tool, a total score of ≥25 has been shown to distinguish anxiety from nonanxiety disorders in clinical and community samples (Birmaher et al., 1999; Canals et al., 2012; Gonzalez et al., 2012).

The Affective Reactivity Index (ARI; Stringaris et al., 2012) is a 7‐item questionnaire used to assess irritability symptoms in children and adolescents. In the current research, we administered both the Self Report (ARI‐S) and Parent Report (ARI‐P) forms, and we assessed symptoms over the past 6 months. Strong psychometric properties of the ARI have been supported in previous research (see Evans et al., 2024 for a review). Items query frequency and severity of irritable mood, frequency of temper outbursts, and overall irritability‐related impairment on a three‐point scale from 0 (*Not True*) to 2 (*Certainly True*). Responses to items 1 through 6 are summed to generate a total score (with item 7, indicating impairment, being considered a separate subscale). Possible scores range from 0 to 12.

#### EMA items

The larger irritability EMA protocol included items focused on children's reports of their irritability and related symptoms (e.g., grouchiness, sadness, temper outbursts, impairment); see the Supporting Information in Naim et al. (2026) for the full EMA item list. The larger anxiety EMA protocol included items focused on various dimensions of mood and anxiety symptoms and their situational context; see Supporting Information S1 in Smith et al. (2019) for the full anxiety EMA item list. The current analyses focused on a subset of four identical EMA items from each protocol relating to symptoms of anxiety and irritability. Two items assessed symptoms of anxiety and broadly align with the structure of the Positive Affect and Negative Affect Schedule for Children (Laurent et al., 1999): “At the time of the beep, I felt worried or scared” and “Since the last beep, I felt worried or scared.” These two anxiety EMA items have demonstrated satisfactory psychometric properties among this sample of youth with anxiety disorders, showing moderate convergent validity with retrospective youth self‐report measures (SCARED‐C) at interval 1 (Byrne et al., 2023). Two items assessed symptoms of irritability and broadly align with items from the ARI (Stringaris et al., 2012): “At the time of the beep, I felt annoyed or angry” and “Since the last beep, I felt frustrated.” Each item was rated on a 5‐point Likert scale from 1 (*Not At All*) to 5 (*Extremely*). These two irritability EMA items have demonstrated satisfactory psychometric properties among a larger sample of youth with irritability, showing moderate convergent validity with retrospective youth self‐ and parent‐report measures (ARI‐S, ARI‐P) (Naim, Smith, et al., 2021).

### Data analytic strategy

#### Missing data

##### Anxiety

Consistent with prior EMA research (Bylsma et al., 2011; Kircanski et al., 2015), useable data was defined as responding to at least 5 of 21 prompts at two of the three time intervals. Of the 152 treatment‐seeking youth with anxiety who were assessed via EMA, we included 92 youth who had useable data for at least two of the time intervals in analyses. Of note, 66 youth had useable data for all three intervals. Youth with anxiety who had at least two useable timepoints (*n* = 92) did not differ from excluded youth with only one useable EMA timepoint (*n* = 60) on age (*t*(113.02) = 0.12, *p* = .90) or sex (*X* ^2^ = 1.56, *p* = .46). Mann‐Whitney U tests indicated that youth with anxiety who had at least two useable timepoints did not differ significantly from excluded youth with only one useable timepoint on any of the four EMA items (*p*s > .71). Independent samples *t* tests indicated that youth with anxiety who had at least two useable timepoints also did not differ significantly from excluded youth on baseline irritability (ARI‐S) or anxiety (SCARED‐C) symptom measures (*p*s > .42). On average, compliance was 71.7% at interval 1 (pre‐treatment), 63.7% at interval 2 (mid‐treatment), and 59.8% at interval 3 (post‐treatment). Compliance in the anxiety sample did not differ by sex at any interval (*p*s > .41) and did not relate to participant age at any interval (*p*s > .51). On average, the latency to respond across all intervals was 14.07 minutes.

##### Irritability

Of the 40 families who completed the treatment protocol, one did not complete EMA at all three intervals, resulting in 39 youth with irritability included in analyses. On average, compliance was 76.60% at interval 1 (pre‐treatment), 72.90% at interval 2 (mid‐treatment), and 65.90% at interval 3 (post‐treatment) (Naim et al., 2025, 2026). Compliance in the irritability sample did not differ by sex at any interval (*p*s > .53) and did not relate to participant age at any interval (*p*s > .15). Of participants with the “response start time” variable (*n* = 17), on average, the latency to respond across all intervals was 16.52 minutes.

##### Healthy volunteers

Seventy‐seven youth with no psychiatric diagnosis participated in EMA. Of those, 57 healthy youth had useable data for at least two of the three timepoints and were included in analyses. Of note, 35 youth had useable data for all three intervals. On average, compliance was 85.5% at interval 1, 75.4% at interval 2, and 74.8% at interval 3. Compliance in the healthy volunteer sample did not differ by sex at any interval (*p*s > .14) and did not relate to participant age at any interval (*p*s > .08). On average, the latency to respond across all intervals was 12.09 minutes.

##### Multilevel models

Due to the nested nature of the data (prompts within intervals within participants), mixed‐effects multilevel modeling was performed in HLM Version 8 (Raudenbush et al., 2019; Snijders & Bosker, 2011). Each analysis included all available timepoints per participant, and missing data were removed via HLM software when running analyses. Level 1 included the within‐subject repeated prompt data, nested within Level 2 which included the within‐subject repeated time intervals (e.g., pre‐, mid‐, post‐treatment), nested within individuals at Level 3 (between‐subjects).

We examined EMA‐rated changes in symptoms for youth with anxiety and healthy volunteers over the course of treatment/over time via mixed‐effects multilevel models, using a parallel data analytic strategy as in Naim et al. (2026) for the sample of youth with irritability. A given symptom item rating for person *j* at interval *i* and prompt *t* (e.g., worry_*tij*_) was predicted by treatment interval (Level 2) entered as a categorical predictor (coded as pre = 0, mid = 0.5, post = 1) and as a function of each participant (Level 3). Including interval at Level 2 allows for estimation of the linear slopes from interval 1 (e.g., pre‐treatment) to interval 2 (e.g., mid‐treatment) to interval 3 (e.g., post‐treatment). The models specified outcome variables as continuous, a random intercept for interval at Level 2, a random intercept for person at Level 3, and a random slope for interval at Level 3. Given Level 2 treatment interval was coded as pre = 0, mid = 0.5, post = 1, the fixed effect of time reflects the model‐implied linear change across the total pre‐to‐post interval.

$$\begin{array}{l} \text{Mixed}\;\text{Model}:{\text{Worry}}_{tij}=\gamma_{000}+\gamma_{010}*{\text{CBT}}_{ij}+r_{0ij}+u_{00j}+u_{01j}*{\text{CBT}}_{ij}+e_{tij}\\ {\text{Level}\;1:\text{Worry}}_{tij}=\pi_{0ij}+e_{tij}\\ \text{Level}\;2:\pi_{0ij}=\beta_{00j}+\beta_{01j}*\left({\text{CBT}}_{ij}\right)+r_{0ij}\\ \text{Level}\;3:\beta_{00j}=\gamma_{000}+u_{00j\;}\\ \beta_{01j}=\gamma_{010}+u_{01j} \end{array}$$

We used the Benjamini–Hochberg procedure for false discovery rate (FDR) correction across all models (*k* = 12), with the expected proportion of false positives set to *q* = 0.05. We report the raw coefficients and standard errors from our multilevel models considering FDR. Supplementary analyses were conducted among a subset of the anxiety sample to test whether their assigned ABMT condition interacted with time to predict symptom outcomes in anxiety or irritability.

## RESULTS

### Sample description

#### Psychological symptoms

At baseline and on average, SCARED‐C (*M* = 30.82, SD = 14.72) and SCARED‐P (*M* = 29.57, SD = 11.98) total scores for youth with anxiety exceeded the ≥25 cutoff indicating elevated anxiety symptoms consistent with a possible anxiety disorder (Birmaher et al., 1999; Canals et al., 2012; Gonzalez et al., 2012). While neither SCARED‐C (*M* = 18.93, SD = 18.74) nor SCARED‐P (*M* = 19.64, SD = 11.69) average total scores for youth with irritability met that threshold, there was marked variability consistent with the presence of anxiety disorders comorbid to primary irritability‐related disorders (DMDD, ODD, or ADHD) in a subset of youth. Youth with anxiety exhibited significantly higher scores on the SCARED‐C (*t*(58.80) = 3.53, *p* < .001) and SCARED‐P (*t*(73.30) = 4.41, *p* < .001) than youth with irritability. For healthy volunteers, average baseline total scores were well below 25 for SCARED‐C (*M* = 6.88, SD = 5.99) and SCARED‐P (*M* = 4.01, SD = 4.64).

At baseline and on average, for the irritability sample, scores for ARI‐S (*M* = 5.33, SD = 3.37) and ARI‐P (*M* = 7.55, SD = 2.68) were consistent with previous research on youth with clinically impairing irritability (Stringaris et al., 2012). For the anxiety sample, scores for the ARI‐S (*M* = 3.74, SD = 3.44) and ARI‐P (*M* = 3.25, SD = 2.81) were consistent with previous research on youth with anxiety disorders (Stoddard et al., 2014). Youth with irritability exhibited significantly higher scores on the ARI‐S (*t*(73.03) = 2.45, *p* = .02) and the ARI‐P (*t*(74.88) = 8.28, *p* < .001) than youth with anxiety. For healthy volunteers, average baseline ARI‐S was *M* = 0.79, SD = 1.08, and ARI‐P was *M* = 0.43, SD = 0.59.

#### EMA

See Table 2 for average EMA‐rated items at each of the three time intervals for each sample. On average, youth with anxiety and irritability reported experiencing each of the clinical symptoms (i.e., momentary anger, frustration, momentary worry, and worry) to a small degree (i.e., between 1.3 and 2 on the 5‐point Likert scale) as measured by EMA. For youth with anxiety, frustration and worry were the most highly endorsed symptoms across time intervals. Worry was significantly higher at interval 1 (e.g., pre‐treatment) among youth with primary anxiety compared to youth with primary irritability, *t*(2, 749) = 7.73, *p* < .001. For youth with irritability, frustration and anger were the most highly endorsed symptoms across all time intervals. Frustration (*t*(2, 749) = 3.91, *p* < .001) and anger (*t*(2, 749) = 7.83, *p* < .001) were both significantly higher at interval 1 among youth with primary irritability compared to youth with primary anxiety. On average, healthy volunteers reported minimal symptoms (i.e., “*not at all*”). Frustration was the most highly endorsed symptom across all time intervals among healthy volunteers.

**TABLE 2:** Descriptive statistics for EMA items by group and interval.

Interval	Anxiety					Healthy volunteer					Irritability				
	M	SD (wp)	ICC (btw)	ICC (wi)	*N* (prompts)	M	SD (wp)	ICC (btw)	ICC (wi)	*N* (prompts)	M	SD (wp)	ICC (btw)	ICC (wi)	*N* (prompts)
“At the time of the beep, I felt worried or scared”															
1	1.46	0.61	0.29	0.71	1385	1.09	0.25	0.07	0.93	994	1.41	0.42	0.48	0.52	626
2	1.46	0.56	0.40	0.60	1172	1.13	0.26	0.13	0.87	902	1.35	0.40	0.62	0.38	589
3	1.43	0.48	0.47	0.53	905	1.10	0.23	0.10	0.90	584	1.30	0.30	0.52	0.48	513
“At the time of the beep, I felt annoyed or angry”															
1	1.42	0.67	0.18	0.82	1385	1.14	0.29	0.16	0.84	994	1.77	0.74	0.36	0.64	626
2	1.41	0.57	0.29	0.71	1172	1.17	0.36	0.12	0.88	902	1.68	0.71	0.43	0.57	589
3	1.40	0.59	0.28	0.72	905	1.16	0.36	0.16	0.84	584	1.51	0.62	0.30	0.70	513
“Since the last beep, I felt frustrated”															
1	1.81	0.80	0.34	0.66	1385	1.30	0.46	0.27	0.73	994	2.01	0.89	0.42	0.58	626
2	1.73	0.72	0.34	0.66	1172	1.32	0.47	0.26	0.74	902	1.79	0.74	0.50	0.50	589
3	1.59	0.63	0.43	0.57	905	1.25	0.37	0.33	0.67	584	1.63	0.63	0.49	0.51	513
“Since the last beep, I felt worried or scared”															
1	1.79	0.72	0.42	0.58	1385	1.10	0.23	0.11	0.89	994	1.46	0.46	0.45	0.55	626
2	1.70	0.63	0.51	0.49	1172	1.14	0.25	0.23	0.77	902	1.35	0.41	0.60	0.40	589
3	1.67	0.61	0.52	0.48	905	1.06	0.15	0.24	0.76	584	1.30	0.28	0.60	0.40	513

*Note*: For irritability and anxiety samples, intervals 1, 2, and 3 corresponded to pre‐, mid‐, and post‐treatment, respectively. For the healthy volunteers, the intervals occurred approximately 5 weeks apart following consent. Means (*M*) are pooled (observation‐weighted) across all valid responses.

Abbreviations: ICC (btw), between‐person intraclass correlation, that is, the proportion of variance due to stable between‐person differences; ICC (wi), within‐person intraclass correlation; that is, the proportion due to within‐person fluctuation across the interval; SD (wp), within‐person standard deviation.

See Supporting Information S1: Figure S1 for EMA item distributions by group and interval.

### Multilevel models

To examine mean levels of EMA‐reported anxiety and irritability symptoms across time intervals, we conducted multilevel models separately for each symptom of interest among each group. Table 3 shows multilevel model results, with unstandardized parameter estimates (PE) referring to the symptom change by treatment phase.

**TABLE 3:** Multilevel model results for EMA‐rated symptoms.

	Irritability (*n* = 39)								Anxiety (*n* = 92)								Healthy volunteers (*n* = 57)							
	Unstd PE	Unstd SE	Std PE	Std SE	*R* ^2^	Slope SD	% reliable Δ	*p*	Unstd PE	Unstd SE	Std PE	Std SE	*R* ^2^	Slope SD	% reliable Δ	*p*	Unstd PE	Unstd SE	Std PE	Std SE	*R* ^2^	Slope SD	% reliable Δ	*p*
Momentary anger	−.28	.10	−.10	.04	.01	.20	74.4	.01^a^	.04	.05	.02	.02	.00	.19	4.4	.43	.04	.04	.03	.03	.00	.24	10.5	.31
Frustration	−.37	.10	−.13	.03	.02	.33	20.5	<.01^a^	−.17	.06	−.06	.02	.01	.35	5.4	.01^a^	.02	.05	.01	.03	.00	.02	33.3	.74
Momentary worry	−.11	.08	−.05	.04	.00	.21	7.7	.15	−.02	.06	−.01	.03	.00	.35	5.4	.75	.02	.03	.01	.03	.00	.07	3.5	.63
Worry	−.16	.09	−.07	.04	.01	.31	7.7	.08	−.08	.06	−.03	.02	.00	.23	.0	.17	−.01	.03	−.01	.03	.00	.02	47.4	.70

*Note*: Standardized (std) and unstandardized (unstd) values presented.

Abbreviations: PE, parameter estimate; SE, standard error.

^a^ Significance survives FDR correction.

We first hypothesized that anxiety patients would exhibit reductions in the targeted symptoms of EMA‐reported worry. This hypothesis was not supported; neither momentary worry nor worry since the last survey changed over the course of CBT for anxiety. In comparison, for youth with irritability, both momentary anger and frustration since the last survey decreased over the course of CBT (Naim et al., 2026).

Second, we hypothesized that non‐targeted symptoms would decrease, such that youth treated for anxiety would demonstrate reductions in EMA‐reported irritability items. This hypothesis was partially supported; momentary anger did not change, while frustration since the last survey decreased over the course of treatment. This result survived FDR correction. In comparison, among youth receiving CBT for irritability, neither momentary worry nor worry since the last survey changed over the course of treatment (Naim et al., 2026).

**FIGURE 1:**
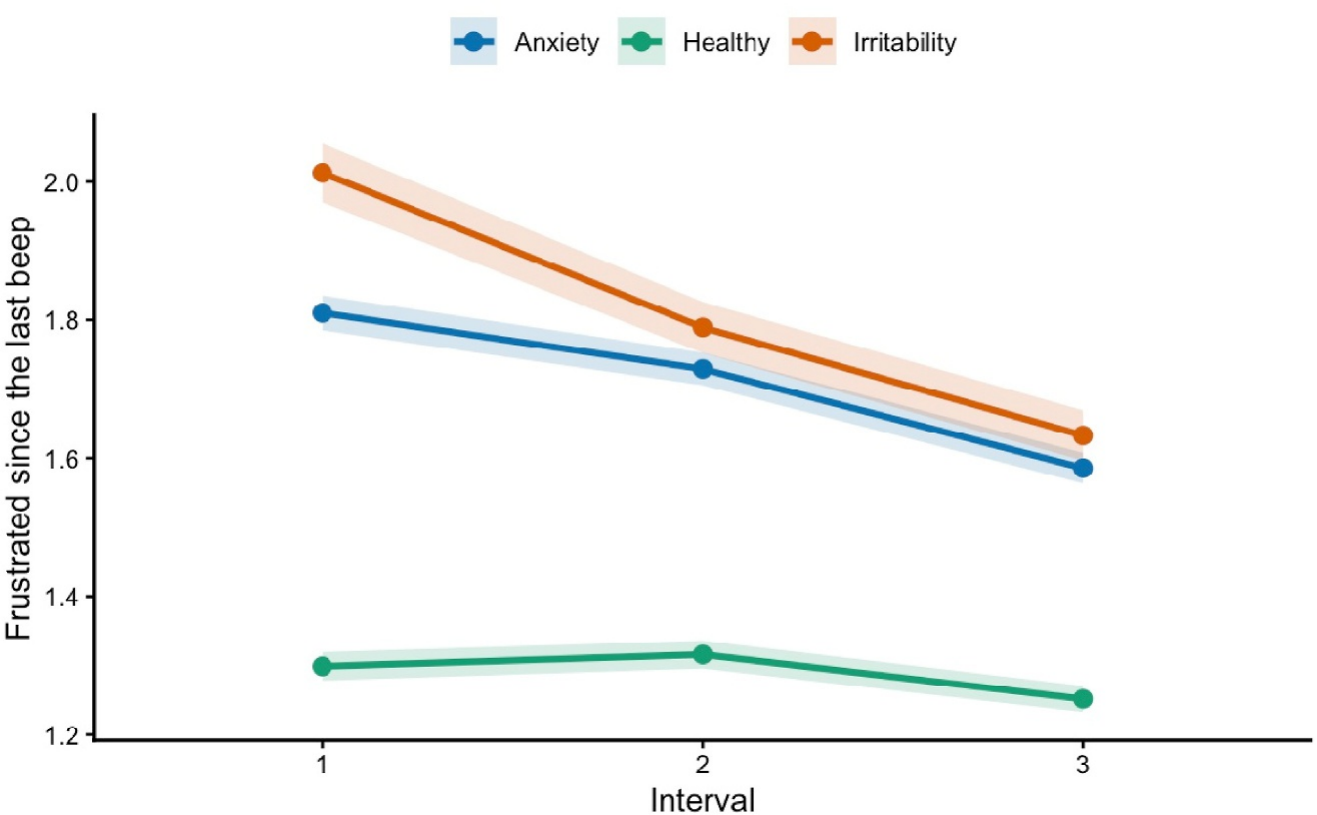
Group differences in EMA‐rated frustration over time. Raw means are presented. Confidence band reflects within‐person standard errors with subject‐level centering.

Lastly, we hypothesized that EMA‐reported symptoms would be low and stable across time among a sample of healthy comparison youth. As expected, among healthy youth, momentary anger and frustration since the last survey did not change over the three time intervals. Similarly, momentary worry and worry since the last survey did not change over the three time intervals for healthy youth. See Figure 1 for a depiction of differences in EMA‐rated frustration across the three time intervals in each group.

For multilevel modeling for youth with anxiety and healthy volunteers, results for youth with two valid time intervals were consistent with results from analyses that included youth who had only one valid time interval and all three valid time intervals. Interpretation of all multilevel model results were consistent in supplementary analyses controlling for age and sex (Supporting Information S1: Table S2). Exploratory analyses examining symptom changes between active versus sham conditions in a subset of youth with anxiety who received ABMT are reported in Supporting Information S1: Table S3. There were no significant interaction effects of ABMT condition (active vs. sham) for any of the symptoms.

## DISCUSSION

### Overview of observed changes in targeted and non‐targeted symptoms in youth receiving CBT for anxiety and irritability

Taking together both the novel findings (youth with anxiety and healthy volunteers) and previously published findings (youth with irritability) from a single, symptom‐focused EMA protocol across distinct but interrelated samples, three key takeaways emerged. First, we observed decreases in some targeted symptoms (i.e., frustration and anger for CBT for irritability) and some non‐targeted symptoms (i.e., frustration for CBT for anxiety), but not others (i.e., worry for CBT for anxiety [targeted] and irritability [non‐targeted]). Second, we observed decreases in frustration among youth receiving both CBT for anxiety and irritability, suggesting that frustration may be a treatment‐relevant transdiagnostic symptom worthy of further study. Third, healthy volunteers showed the expected pattern of low, stable symptoms, underscoring that the EMA items captured clinically meaningful symptom variation and that symptom decreases among clinical youth reflected reduction rather than normalization.

### Observed decreases in frustration among youth receiving CBT for anxiety and irritability

As reported in Naim et al. (2026), we observed decreases in frustration and anger in youth receiving CBT for irritability, which partially supports that patients exhibit targeted reductions in in vivo symptoms corresponding to their primary diagnoses. We also observed decreases in frustration among youth receiving CBT for anxiety, although frustration was not an explicit target of the anxiety protocol. Of note, these EMA findings are consistent with our previous work finding CBT for irritability leads to decreased irritability via blinded clinician, parent, and child reports (Naim et al., 2024), and CBT for anxiety is associated with decreased irritability via child report (see Byrne et al., 2026 Supplement), underscoring the convergent validity of EMA with these measures (Hansen et al., 2025).

Our finding that frustration improved in youth receiving two versions of exposure‐based CBT—one tailored to irritability and one tailored to anxiety—highlights the transdiagnostic nature of frustration. Indeed, previous research identified frustration as a central node in between‐ and within‐subject network analysis of youth with clinically impairing irritability and anxiety disorders; feeling more frustrated was associated with feeling more worried contemporaneously, and feeling more frustrated at one timepoint predicted changes in mood at the subsequent timepoint (Tseng et al., 2023). It is possible that by targeting and reducing frustration (e.g., decreasing frustration or increasing tolerance for experiencing it), interventions may also reduce associated symptoms key to irritability (e.g., anger) and anxiety (e.g., worry; Tseng et al., 2023). Therefore, frustration warrants further study as a potential treatment target for both CBT for anxiety and irritability (Stoddard et al., 2014), with implications for the development of transdiagnostic pediatric mental health treatments. A critical next step is to test this possibility directly by examining whether changes in frustration mediate targeted symptom improvement. Lagged EMA designs may help clarify the temporal coupling between irritability and other symptoms.

### No observed changes in worry among youth receiving CBT for anxiety or irritability

Our finding that in vivo worry did not change over time among youth with anxiety is contrary to our hypothesis that these patients would exhibit reductions in targeted symptoms corresponding to their primary diagnoses. Further, this finding is contrary to our previous work demonstrating decreases in child‐ and clinician‐rated anxiety over the course of CBT for anxiety (Byrne et al., 2026). Previously, we found satisfactory psychometric properties of these EMA items among youth with anxiety disorders, demonstrating moderate convergent validity with retrospective child report measures (SCARED‐C) but not with parent (SCARED‐P) or clinician report (i.e., Pediatric Anxiety Rating Scale) at interval 1 (Byrne et al., 2023). This pattern may partially reflect shared informant variance, and it is consistent with known informant discrepancies in youth psychopathology (De Los Reyes & Epkins, 2023). While it is possible that diverging symptom reports between children and their parents/clinicians may yield unique and valuable information about children's anxiety, and future research should consider collecting and interpreting EMA data from multiple reporters, it is also possible that our worry EMA items did not adequately capture the lived experience of children's anxiety in real time. Although there was variability, mean ratings were lower than expected among youth with anxiety disorders (Byrne et al., 2023). Anxiety is multifaceted. Our items designed to capture anxiety queried children's feelings of being “worried or scared,” which may capture two components of anxiety. There are other cognitive (e.g., difficulty concentrating), emotional (e.g., nervousness), behavioral (e.g., avoidance), and physiological (e.g., increased heart rate) components uncaptured in this phrasing. Researchers should consider including additional items and co‐designing items with patients to better capture their experiences (Clark et al., 2025; Metcalf et al., 2025). Finally, it is possible that frustration represents a more proximal and shared affective target of CBT across anxiety and irritability presentations than worry. In both CBT manuals, youth are exposed to situations that elicit distress and are challenged to refrain from prepotent maladaptive responses. These tasks, and the process of therapy more generally, may be frustrating for youth and implicitly or explicitly require them to tolerate it. Accordingly, changes in EMA‐rated frustration may have preceded changes in EMA‐rated worry (Tseng et al., 2023), and our design could not capture those changes in worry due to the lack of a fourth interval. Future EMA research would benefit from follow‐up intervals to capture temporal unfolding and mechanisms of symptom change in the context of psychotherapy across disorders.

### No observed symptom changes in healthy volunteers over time

We included a sample of healthy volunteer youth to compare EMA item levels and trajectories over time relative to our clinical samples. As expected, healthy volunteers had lower levels of symptoms at baseline compared to youth with anxiety and irritability that remained low over the three assessment intervals (matched for time to be consistent with the pre‐, mid‐, and post‐treatment timepoints of CBT). Further, healthy volunteers demonstrated less variability in responding compared to youth with anxiety and irritability at each interval. Healthy volunteers showed no changes in their in vivo symptom reports over time. Although EMA‐reported symptoms decreased over time among youth with anxiety and irritability, their reports remained higher and more variable than those of healthy volunteers at the final assessment. Taken together, EMA items captured meaningful differences in symptom level and variability between clinical and healthy youth, with clinical youth showing symptom reduction over time rather than normalization. Future research would benefit from additionally including clinical, treatment‐seeking comparison samples to examine whether symptom reductions in youth receiving CBT for anxiety and irritability were attributable to the effect of self‐monitoring symptoms by completing surveys multiple times per day (i.e., measurement reactivity; Barta et al., 2012).

### Strengths and limitations

Our study had several methodological strengths. Research leveraging EMA to evaluate psychotherapy outcomes is rare (Mink et al., 2025). To our knowledge, ours is one of the largest samples of treatment‐seeking youth undergoing psychotherapy and completing EMA across two different primary diagnoses. EMA facilitated measurement of these dynamic symptoms multiple times per day in a natural environment, thus minimizing recall bias and improving ecological validity. Parallel EMA protocols allowed us to compare symptom changes across groups. Unlike most existing pediatric EMA research, this study included a healthy comparison sample to support interpretation of EMA‐reported symptoms in the clinical samples.

This study had limitations that are worth discussion. First, while it was expected that in vivo anxiety and irritability symptoms would be less marked among healthy individuals than clinical samples, there were low mean ratings and limited variability for item responses among the healthy group. Further, the range of responses for targeted and non‐targeted symptoms among the groups with anxiety and irritability was lower than expected, which may indicate a measurement limitation that negatively impacted the ability to detect changes in worry or anger items over time. Researchers might consider using continuous visual analog scales to improve variability (Haslbeck et al., 2025). Second, only a subset of the anxiety sample was assigned to the ABMT condition, which might dilute the treatment effects for anxiety or obscure to which aspects of treatment they might be attributed. However, in supplementary analyses, ABMT did not interact with time to predict symptom outcomes in anxiety or irritability. Third, across the study protocols of CBT for anxiety and irritability, there were differences in what constituted pre‐, mid‐, and post‐treatment timepoints. In CBT for anxiety, EMA was assessed prior to the beginning of exposures at session 2, between sessions 7 and 8, and between sessions 11 and 12. In CBT for irritability, EMA was assessed prior to CBT session 1, between sessions 6 and 7, and after session 12. The abbreviated timescale in CBT for anxiety may have limited the ability to observe in vivo symptom changes in worry across treatment. Future research might consider administering EMA entirely before and after treatment, as well as at follow‐up (e.g., 1, 3, and/or 6 months). Fourth, as mentioned above, the mechanisms of change that could be driving the link between anxiety‐focused CBT and reductions in frustration are unclear and should be tested through mediation analyses in future research. For example, anxiety and irritability psychoeducation embedded in each respective treatment protocol may have resulted in children's improved recognition and regulation of targeted and non‐targeted emotions. Of note, it is possible that enhanced emotion recognition may have also disrupted EMA measurement, such that increased awareness and reporting of irritable and anxious emotions over treatment may have attenuated observed symptom reductions in the irritability sample and contributed to the null findings in the anxiety sample. Therefore, future treatment studies would benefit from measuring changes in emotion recognition directly and supplementing child‐reported EMA with other measures of symptom change (e.g., parent‐reported EMA, clinician ratings). Lastly, the youth samples were skewed homogenous in demographic characteristics, which may limit the generalizability of these findings to youth of other backgrounds than the current samples.

## CONCLUSION

In summary, in a large, longitudinal analysis of youth's in vivo symptom changes over the course of tailored CBT, we observed decreases in in vivo frustration among both anxiety and irritability samples. CBT targeting anxiety may have implicit effects on non‐targeted symptoms related to irritability, such as frustration. Results provide a foundation for future studies to more precisely characterize shared and distinct symptom processes in youth with anxiety and irritability, and ultimately inform shared and precise symptom targeting for youth undergoing CBT for anxiety and irritability.

## AUTHOR CONTRIBUTIONS

**Lauren M. Henry**: Conceptualization; writing—original draft; writing—review and editing; methodology; formal analysis; data curation; project administration; validation. **Meghan E. Byrne**: Conceptualization; methodology; formal analysis; writing—original draft; writing—review and editing; data curation; project administration; visualization; validation. **Julia Modell**: Conceptualization; formal analysis; writing—original draft; writing—review and editing. **Lili Massac**: Conceptualization; formal analysis; writing—original draft; writing—review and editing. **Krystal Lewis**: Conceptualization; writing—review and editing; methodology. **Simone Haller**: Writing—review and editing; formal analysis; methodology. **Reut Naim**: Conceptualization; methodology; data curation; formal analysis; writing—review and editing; project administration; validation. **Katharina Kircanski**: Conceptualization; methodology; formal analysis; writing—review and editing. **Daniel S. Pine**: Conceptualization; methodology; supervision; funding acquisition; writing—review and editing; resources; project administration. **Melissa A. Brotman**: Conceptualization; methodology; supervision; funding acquisition; writing—original draft; writing—review and editing; resources; project administration.

## CONFLICT OF INTEREST STATEMENT

The authors declare no conflicts of interest.

## ETHICS STATEMENT

Informed consent and assent have been appropriately obtained for all participants. The protocol for the anxiety and healthy volunteer samples was initially approved by the National Institute of Mental Health Institutional Review Board on May 25, 2001 (IRB #: 01M0192). The protocol was most recently approved via a continuing renewal on May 28, 2025 (IRB #: 01M0192/CR003134, Federalwide Assurance # FWA00005897). The protocol for the irritability sample was approved by the National Institute of Mental Health Institutional Review Board (IRB #: 02‐M‐0021, iRIS Reference #: 559333, EMA Approval Date: June 29, 2021; IRB #: 15‐M‐0182, Initial Approval Date: June 30, 2015).

## Supporting information

Supporting Information S1

## Data Availability

For the irritability sample, data for those who consented to share are publicly available on OSF (osf.io/khmya). For the anxiety and healthy volunteer samples, data for those who consented to share are publicly available on OSF (osf.io/r89zp). Given not all participants consented to share their data, exact replication of analyses with the open dataset will not be possible.
