# Supplementary material for: Ecological momentary assessment of targeted and non‐targeted symptom changes in clinical anxiety and irritability among children and adolescents receiving cognitive behavioral therapy
Source: JCPP Adv. 2026 Aug 30:e70160. Online ahead of print. doi: 10.1002/jcv2.70160 (PMC13527456; doi:10.1002/jcv2.70160)
Supplement: Supplementary file 1 [file JCV2-9999-e70160-s001.docx]

**Ecological Momentary Assessment of Targeted and Non-Targeted Symptom Changes in Clinical Anxiety and Irritability among Children and Adolescents Receiving Cognitive Behavioral Therapy**

**Supporting Information**

**Appendix S1.** Sample Comorbidity

*Anxiety Sample.* Three youth (3.26%) also met criteria for a comorbid diagnosis of attention-deficit/hyperactivity disorder (ADHD). No youth with a primary anxiety disorder in the current sample met criteria for a comorbid diagnosis of oppositional defiant disorder (ODD) or disruptive mood dysregulation disorder (DMDD).

*Irritability Sample.* In the primary DMDD group, 19 youth (79.17%) met criteria for a comorbid diagnosis of ADHD, 12 (50.00%) for generalized anxiety, 4 (16.67%) for separation anxiety, 4 (16.67%) for a specific phobia, 2 (8.33%) for chronic motor disorder, 1 (4.17%) for social phobia, 1 (4.17%) for a tic disorder, 1 (4.17%) for enuresis, and 1 (4.17%) for excoriation disorder. In the primary ODD group, 5 youth (50.00%) met criteria for a comorbid diagnosis of ADHD, 3 (30.00%) for separation anxiety, and 2 (20.00%) for generalized anxiety. In the primary ADHD group, 2 (40.00%) youth met criteria for a comorbid diagnosis of enuresis, 1 (20.00%) for separation anxiety, 1 (20.00%) for generalized anxiety, and 1 (20.00%) for social phobia.

Table S1

Adapted STROBE Checklist for Reporting EMA Studies (CREMAS)

| **Section and Topic** | **Item #** | **Checklist Item** | **Section and page number reported** |
| --- | --- | --- | --- |
| **TITLE** | | | |
| Title | 1 | Include ecological momentary assessment in title and key words | Title (pages 1 and 2) |
| **INTRODUCTION** | | | |
| Rationale | 2 | Briefly introduce the concept of EMA and provide reasons for utilizing EMA for this study or topic of interests (e.g., to examine time-varying predictors of unhealthy eating occasions in children’s daily lives) | Introduction (page 2) |
| **METHODS** | | | |
| Training | 3 | Indicate if, and by what methods, training of participants for EMA protocol was used | Methods (page 4) |
| Technology | 4 | Describe what technology, if any, was used. Include the following information: device (e.g., mobile phone, portable computer), model (e.g., Nexus 4, iPod), operating system (e.g., Android, Windows), and EMA program name | Methods (page 4) |
| Wave Duration | 5 | State the number of waves for the study (e.g., 2 monitoring periods over the course of 1 year) | Methods (page 4) |
| Monitoring Period | 6 | State the number of days each wave of the study lasted, and how many weekdays versus weekend days | Methods (page 4) |
| Prompting Design | 7 | Indicate the prompting strategy used for the study (e.g., event-based, interval-based, or a combination of the two). If using interval-based strategy, indicate what type of schedule is used (e.g., fixed, random, or hybrid interval) | Methods (page 4) |
| Prompt Frequency | 8 | Intended frequency of prompts per day. Break down by weekdays and weekend days if applicable | Methods (page 4) |
| Design Features | 9 | Describe any design feature to address potential sources of bias (e.g., reactivity) or participant burden (e.g., EMA questions appearing in different orders) | NA |
| **RESULTS** | | | |
| Attrition | 10 | Indicate participant attrition throughout the study; report attrition rates both by monitoring days and waves, if applicable | Methods (pages 5 and 6) |
| Prompt delivery | 11 | Report number of EMA prompts that were planned to be delivered. If possible, also report the number of EMA prompts that were actually received by participants and indicate reasons for why prompts were not sent out (e.g., technical issues or participant noncompliance reason such as phone was powered off) | Methods (page 4) |
| Latency | 12 | Report the amount of time from prompt signal to answering of prompt | Methods (pages 5 and 6) |
| Compliance Rates | 13 | Report total answered EMA prompts across all subjects and the average number of EMA prompts answered per person. Report compliance rate both by monitoring days and waves, if applicable. Indicate reasons for noncompliance, if known | Methods (pages 5 and 6) |
| Missing Data | 14 | Report whether EMA compliance is related to demographic or time-varying variables | Methods (pages 5 and 6) |
| **DISCUSSION** | | | |
| Limitations | 15 | Discuss limitations of the study, taking into account sources of potential bias when using EMA methods (e.g., reactivity, use of technology) | Discussion (pages 9 and 10) |
| Conclusions | 16 | Provide a general interpretation of results and discuss the benefits of using EMA (e.g., improving understanding of daily behaviors) | Discussion and Conclusion (page 10) |

*From:* Liao, Y., Skelton, K., Dunton, G., & Bruening, M. (2016). A systematic review of methods and procedures used in ecological momentary assessments of diet and physical activity research in youth: An adapted STROBE checklist for reporting EMA studies (CREMAS). *Journal of Medical Internet Research, 18*(6), e151. <https://doi.org/10.2196/jmir.4954>

Table S2

Multilevel Model Results for EMA-rated Symptoms, Adjusting for Age and Sex

|  | Irritability  (*n =* 39) | | | | | Anxiety  (*n =* 92) | | | | | Healthy Volunteers  (*n =* 57) | | | | |
| --- | --- | --- | --- | --- | --- | --- | --- | --- | --- | --- | --- | --- | --- | --- | --- |
|  | Unstd PE | Unstd SE | Std PE | Std SE | *p* | Unstd PE | Unstd SE | Std PE | Std SE | *p* | Unstd PE | Unstd SE | Std PE | Std SE | *p* |
| Momentary Anger | -0.28 | 0.10 | -0.10 | 0.04 | .01^†^ | 0.04 | 0.05 | 0.02 | 0.02 | .41 | 0.04 | 0.04 | 0.03 | 0.03 | .31 |
| Frustration | -0.37 | 0.10 | -0.13 | 0.03 | <.01^†^ | -0.16 | 0.06 | -0.06 | 0.02 | .01^†^ | 0.01 | 0.05 | 0.01 | 0.03 | .77 |
| Momentary Worry | -0.11 | 0.08 | -0.05 | 0.04 | .15 | -0.02 | 0.06 | -0.01 | 0.03 | .76 | 0.01 | 0.03 | 0.01 | 0.03 | .70 |
| Worry | -0.16 | 0.09 | -0.07 | 0.04 | .07 | -0.08 | 0.06 | -0.03 | 0.02 | .18 | -0.01 | 0.03 | -0.01 | 0.03 | .66 |

*Note*. ^†^ Significance survives FDR correction. Multilevel models adjusted for age and sex as fixed person-level covariates. PE = parameter estimate. SE = standard error. Standardized (std) and unstandardized (unstd) values presented.

Table S3

Multilevel Model Results for the ABMT-by-Time Interaction on EMA-rated Symptoms in Youth with Anxiety who Completed ABMT

(*n* = 90)

|  | Unstd PE | Unstd SE | Unstd 95% CI | Std PE | Std SE | Std 95% CI | *df* | *p* |
| --- | --- | --- | --- | --- | --- | --- | --- | --- |
| Momentary Anger | 0.04 | .09 | -0.15, 0.22 | 0.02 | .04 | -0.07, 0.11 | 88 | .70 |
| Frustration | 0.07 | .12 | -0.17, 0.31 | 0.03 | .05 | -0.06, 0.12 | 88 | .56 |
| Momentary Worry | -0.06 | .11 | -0.28, 0.17 | -0.03 | .05 | -0.13, 0.08 | 88 | .62 |
| Worry | -0.20 | .12 | -0.44, 0.03 | -0.07 | .04 | -0.16, 0.01 | 88 | .09 |

*Note.* ABMT = Attention bias modification training. PE = parameter estimate. SE = standard error. CI = confidence interval. Standardized (std) and unstandardized (unstd) values presented. Sample includes all youth with anxiety who completed ABMT and had at least two useable EMA timepoints.

Figure S1. EMA Item Distributions by Group and Interval

*Note*. Density plots by group and interval are depicted.
